# Career expectations and the motivating factors for studying dentistry in Libya

**DOI:** 10.1186/s12909-022-03933-3

**Published:** 2022-12-07

**Authors:** Niroz Arhoma, Maha El Tantawi, Arheiam Arheiam

**Affiliations:** 1 Department of Dental Public Health and Preventive Dentistry, Faculty of Dentistry, University of Benghazi, Benghazi, Libya; 2 Department of Pediatric Dentistry and Dental Public Health, Faculty of Dentistry, Alexandria University, Alexandria, Egypt

**Keywords:** Motivations, Career expectations, Dental students, Libya

## Abstract

**Introduction:**

This study investigated entry-level dental students’ motives for studying dentistry at the University of Benghazi (UoB), Libya and career expectations among recent UoB graduates in an atypical context during the time of political unrest and identified factors associated with these motives.

**Methods:**

A cross-sectional survey was conducted in 2021–2022 for all entry-level dental students and recent dental graduates of UoB. A self-administered survey explored motives for studying dentistry, career expectation and associated factors. The questions were adopted from previous studies and pre-validated for use among Libyan students. Motives and career expectations were summarized, and their association with potential associated factors were assessed using chi-square test at *p* ≤ 0.05.

**Results:**

One hundred eighty-four entry-level students and 156 recent graduates completed the surveys, response rates = 73.6% and 62.4%, respectively. The main motives to study dentistry were the desire to work in the healthcare field (183, 99.4%), interest in scientific knowledge (178, 96.7%) and because there were various dental specialities (168, 91.3%). The most common career expectations were setting up a business (107, 68.6%) and establishing a dental clinic (105, 67.3%). In addition, preference for working in the public sector (106, 67.9%), work-life balance (102, 65.4%) and financial gain (94, 60.3%) were the main factors associated with career expectations.

**Conclusion:**

The main motive to join a dental school in Libya was academic interest. However, recent graduates showed more pragmatic expectations related to private practice ownership.

## Introduction

Motives are the drivers to do something or pursue a goal. They can be intrinsic, reflecting the innate human drive to learn and adapt, or extrinsic, affected by external control and goals [1]. Some psychologists portray motivation as a complex, multifaceted construct consisting of curiosity, positive self-regard, fear and power [2, 3]. Motives have a well-documented role in the educational process, shaping career choices, influencing the academic journey towards competence, and affecting job satisfaction [4, 5]. Motives are important in pursuing any career, and studies have shown that dentistry has unique motives and drives [6]. For instance, previous studies have suggested that dental students emphasize the importance of financial gain in choosing dentistry as a career [7], unlike medical students who prioritize humanitarianism [8]. Moreover, the motives to study dentistry vary considerably among countries over time and according to social, cultural and economic backgrounds [4, 9–11]. On the other hand, career choices and expectations are not always made under the best conditions.

A range of personal, economic and environmental factors may influence the pursuit of career goals [12]. The social unrest and political instability in the Middle East and North Africa (MENA) region have been linked to extreme stress on the labor market and increased unemployment levels, even among highly educated individuals [13]. Since 2011, Libya, a North African country, has experienced political and economic unrest [14]. According to current Libyan government sources, the number of dental schools, dental students, and graduates has surged in recent years at the expense of quality and without considering the country’s actual needs [15]. There is no empirical evidence on motives to study dentistry or career expectations in war-torn Libya. Therefore, the current study explores the motives for studying dentistry, career expectations and associated factors among Libyan dental students.

## Methods

Ethical approval for this study was obtained from the Research Ethics Committee at the Libyan Association of Dental Research (Ref: LADR-061). Participation in the study was voluntary after providing informed consent. The study was conducted according to relevant guidelines and the Helsinki Declaration.

### Design and setting

A cross-sectional validated self-administered survey was distributed to entry-level students and recent UoB graduates in 2020–2021. UoB is the only governmental university in Benghazi and it has the oldest and largest dental school in Libya [14]. The dentistry program at UoB is traditional and comprised of premedical years, two pre-clinical years, two clinical years and a final internship year. Participants were included if they were entry-level students who had recently completed their pre-dental year (*n* = 420) and recent graduates who had completed their internship training (*n* = 370). All students and graduates were invited to participate.

### Data collection tool

Two questionnaires were used in this study. The first questionnaire explored motives to study dentistry among entry-level students. Another questionnaire assessed career expectations among recent dental graduates. Both questionnaires were translated into Arabic and validated for relevance before the study. No modifications were needed for the translated version; hence, the pre-tested questionnaires were used in the study. Each questionnaire took an average of 20 min to complete.

The motives questionnaire was adopted from a previous study conducted in Malaysia [11]. It was divided into two sections. The first section included questions about the participants’ sociodemographic characteristics and potential factors influencing their motives to study dentistry (age, sex, and whether the students have relatives in dentistry). The second section included statements that describe motives to study dentistry, with responses on a five-point Likert scale ranging from ‘strongly disagree’ (score 0) to ‘strongly agree’ (score 4).

The career expectations questionnaire was adopted from a previous study conducted among dental graduates in Malaysia [16]. It was divided into three sections. The first section collected sociodemographic information. The second and third sections explored short-term career expectations and their influencing factors. The responses to items in sections two and three were recorded on a five-point Likert scale ranging from strongly disagree (score 0) to strongly agree (score 4).

The surveys were distributed by the registrar’s office to include as many potential participants as possible, even though not all eligible students visited the registrar during this survey interval. The motives survey was administered to entry-level students while completing their registration. The career expectations survey was administered to recent dental graduates while submitting their documents to obtain the graduation certificate. A research assistant, trained to collect data, was present at the time of survey distribution to answer the queries of the participants. Participation in the study was voluntary, and consent was implied by returning a completed survey to the registrar’s office.

### Statistical analysis

The Statistical Package for the Social Sciences (SPSS 25) was used for analysis. Descriptive analysis was undertaken to summarize the distribution of participants’ characteristics and the distribution of different motives, career expectations and influencing factors. The responses to motives statements were dichotomized as positive responses (agree and disagree) and negative ones (strongly disagree, disagree, and indifferent). Chi-square test was used to assess associations between motives and sociodemographic characteristics of respondents. All statistical tests were conducted with significance set at a *p*-value of 0.05.

## Results

Responses were obtained from 184 to 273 entry-level students and 156 of 243 recent graduates (response rates = 67.4% and 64.2%). Most respondents were females (entry-level: 139, 75.5%, recent graduates: 133, 85.3%), and more than half of their mothers (entry-level: 110, 59.8%, recent graduates: 83, 53.2%) and fathers (entry-level: 99, 53.8%, recent graduates: 97, 62.2%) had a university or higher education. In addition, 71 (38.6%) of entry-level students and 40 (25.6%) of recent graduates had a relative working in dentistry (Table 1).

**Table 1:** Sociodemographic characteristics of entry-level students (*n* = 184) and recent graduates (*n* = 156)

Variable		Entry-level students		Recent graduates	
		N	%	N	%
**Sex**	Male	45	24.5	23	14.7
	Female	139	75.5	133	85.3
**Father education**	University or higher	99	53.8	97	62.2
	Less than University	85	46.2	59	37.8
**Mother education**	University or higher	110	59.8	83	53.2
	Less than University	71	38.6	73	46.8
**Have a relative in dentistry**	Yes	71	38.6	40	25.6
	No	110	59.8	116	74.4

**Fig. 1:**
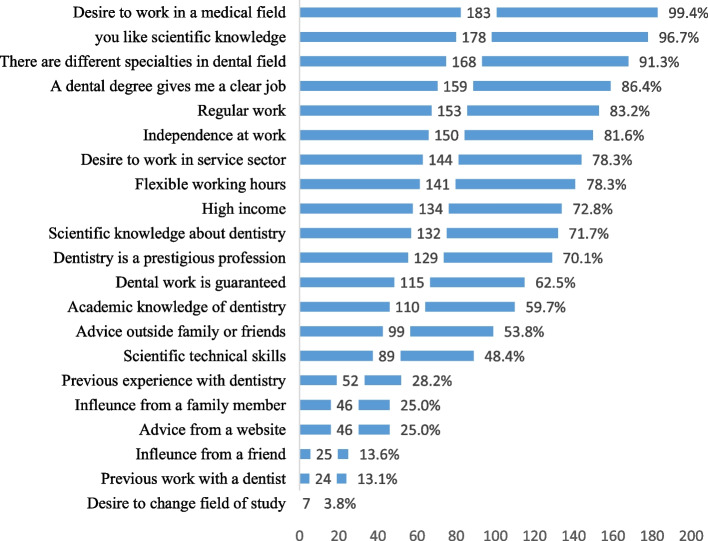
Percentage of respondents who agreed/ strongly agreed on motives to study dentistry

Figure 1 depicts the proportions of entry-level students who agreed and strongly agreed that the statements represent their motives to study dentistry. The most common motives were the desire to work in the healthcare field (183, 99.4%), interest in scientific knowledge (178, 96.7%), and realizing that there are distinct dental specialities (168, 91.3%). Few participants indicated that their motive was the influence of peers (25, 13.60%), observing other dentists (24, 13.1%), and desire to change the field of study (7, 3.8%).

Statistically significant associations were observed between the sociodemographic attributes of entry-level students and some motives to study dentistry. A significantly higher percentage of males was motivated by the desire to work in the healthcare field (*p* = 0.032) and the prestige of dentistry as a profession (*p* = 0.010). Students whose relatives work in the dental field were more likely to be motivated by observing other dentists (*p* = 0.041) and academic knowledge (*p* = 0.014). Higher proportions of dental students with parents with less than a university education, were motivated by advice from a dentistry-related websites (fathers’ education, *p* = 0.019 and mothers’ education = 0.038, Table 2).

**Table 2:** Association between some motives and sociodemographic characteristics of entry-level students

Motives	**Sociodemographic characteristics**	**Motivation responses** **N (%)**		***P* value of X**^**2**^** test**
		**Positive response**	**Negative response**	
**Desire to work in the healthcare field**	Male	26 (57.8)	19 (42.8)	0.032
	Female	55 (39.4)	84 (60.6)	
**Dentistry is a prestigious profession**	Male	30 (66.7%)	15 (33.3%)	0.010
	Female	62 (44.6%)	77 (55.4%)	
**Academic knowledge of dentistry**	Have relatives who work in Dentistry	44 (62%)	27 (38.0%)	0.014
	No relatives work in Dentistry	49 (43.3%)	64 (56.6%)	
**Observing other dentists**	Have relatives working in Dentistry	21 (29.6%)	50 (70.4%)	0.041
	No relatives working in Dentistry	19 (16.8%)	94 (83.2%)	
**Advice from a dentistry-related website**	Father has a university degree or higher	14 (14.1%)	85 (85.9%)	0.019
	Father has less than a university degree	24 (28.2%)	61 (71.8%)	
	Mother has a university degree or higher	17 (15.5%)	93 (84.5%)	0.038
	Mother has less than a university degree	20 (28.2%)	51 (71.8%)	

**Fig. 2:**
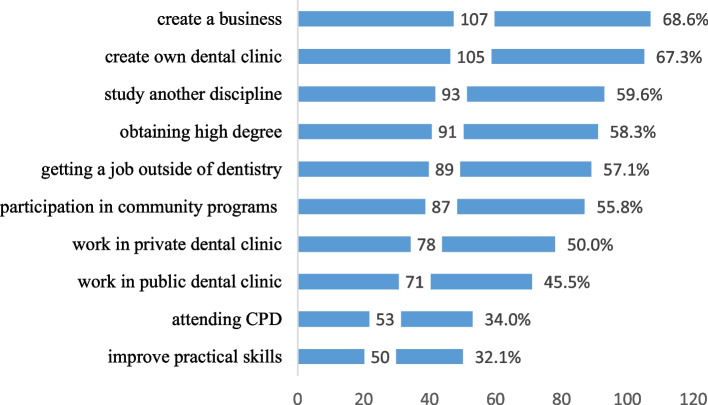
Career expectations among recent graduates

The most common career expectations were setting up a business (107, 68.6%), establishing private practice (105, 67.3%), and studying another discipline (93, 59.6%). On the other hand, continuing professional development (CPD) (53, 34%) and improving practical skills (50, 32.1%) were the least common career expectations (Fig. 2).

**Fig. 3:**
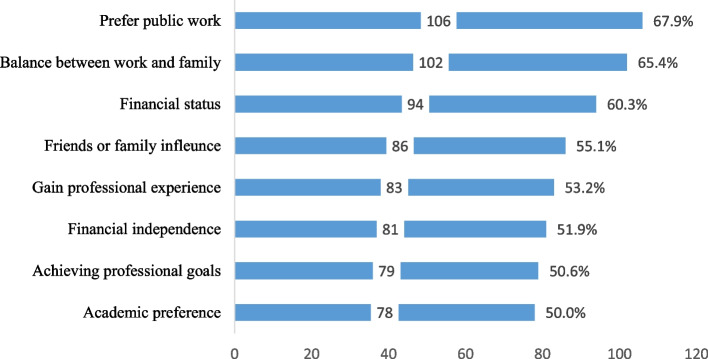
Factors influencing career expectations among recent graduates

Preferring to work in the public sector (106, 67.9%), work-life balance (102, 65.4%) and financial gain (94, 60.3%) were the most common influencers of career expectations, whereas achieving professional goals (79, 50.6%) and academic preference (78, 50%) were least common (Fig. 3).

## Discussion

This study indicates that the motives for studying dentistry among entry-level students in Libya were related to the nature of dentistry as a profession, including working in the healthcare field, scientific knowledge, and realizing that there were different specialties in dentistry. These findings are consistent with previous studies conducted in African countries that showed the altruistic nature of motives to study dentistry [17, 18]. However, variations in the motives to study dentistry in different societies and countries are well-documented [19].

Income was not a primary motive to study dentistry for Libyan dental students which disagrees with studies conducted in other Arab countries showing that income was a strong motive [20, 21]. This may be because the students at that age are financially dependent on their families and most respondents were females for whom the financial motives may be of less concern in the Libyan society. In previous studies, women were more motivated by flexible working hours and altruism than men, indicating that financial freedom and self-employment were primary motives for studying dentistry [22–24]. In this study, males were more likely to be motivated by prestige and working in the healthcare field. The admission of more women in dental schools emphasizes the gender differences in the motives to study dentistry which have been reported previously where women tend to have more concerns about work-life balance [25]. The current trends in Libya indicate an increase in the number of female dentists and this may be associated with further differences in career choices and their motives. Further research is needed to understand why there is an increase in women who study dentistry and other health professions in the Libyan population.

In this study, family attributes seemed to influence the motives to study dentistry. Students whose parents had less than a university education were more likely to be motivated by advice from a dentistry-related website. A possible explanation for this might be that parents with less education may not have adequate skills to advise their offsprings about dental career choices, so the students turn to the internet for information about dentistry. In the present study, those with relatives in the dental field were more likely to be motivated by observing other dentists and academic knowledge. Previous studies reported similar observations in Japan, Iran, and Germany [19, 22, 25]. These findings agree with studies showing the family’s influence on career choices [23, 26] which may be expected since most students live with their families when deciding on their careers [27]. Increasing parental awareness and student career options may mitigate ill-informed decisions regarding choosing dentistry as a future career.

The most common career expectations were setting up private practices and establishing a business. Most recent Libyan dental graduates are self-employed, and a small proportion of them work for fixed salaries in the public sector [15]. By contrast, studies of European dental students reported that they would not like to establish their practice until several years after their training [28] reflecting variations in the healthcare systems. Experts in Libya called for reforming the healthcare system to accommodate the emerging workforce which exceeds the actual capacity of the health sector [29]. Another common career expectation among recent Libyan dental graduates was joining postgraduate studies. Similar findings in Nigeria, Saudi Arabia and Iran indicated that engaging in postgraduate studies was the first career preference among dental students [18, 20, 30]. The preference for part-time governmental jobs, family-work balance and financial gain were the most common influencers of career expectations in this study. Similar findings were observed in previous studies conducted in Malaysia, U.K. and UAE [16, 31, 32]. These findings may reflect the increased admission of women in dental schools and the increasing number of women joining the workforce due to the changing role of women in society. A similar phenomenon was observed in other professions such as medicine [33].

This study is the first of its kind in Libya. However, there are some limitations which need to be acknowledged. First, the study used a cross-sectional design that only provides a snapshot of the situation rather than actual change. The study can, however, serve as a baseline for longitudinal follow-up. Second, the study used self-administered surveys which help collect data from a large population, although this may carry the risk of low response rates [34]. However, the response rate in this study was reasonable [35]. Third, the study was conducted in one site only and thus, the results cannot be generalized to the whole country. However, Benghazi is the largest and oldest dental school in Libya, and it has students from different parts of the country, increasing the sample representativeness. Unfortunately, the war makes the inclusion of several dental schools difficult, if not impossible.

## Conclusion

This study demonstrated that working in the healthcare field and scientific knowledge were the main motives to study dentistry among entry-level Libyan dental students. Some motives were associated with gender, and parental education, highlighting the need to increase awareness about career options among parents as key players in decision-making. Recent Libyan graduates have high expectations of setting up private practices, establishing a business and joining postgraduate studies after graduation. Career expectations were influenced mainly by the preference for working in the public sector, work-life balance and financial gain.

## Data Availability

The datasets generated and analyzed in this study are not publicly available due to restrictions imposed by the Research ethics committee at UoB. The datasets can only be available from the corresponding author on reasonable request.
